# Targeting the Mannose Receptor with Functionalized Fucoidan/Chitosan Nanoparticles Triggers the Classical Activation of Macrophages

**DOI:** 10.3390/ijms24129908

**Published:** 2023-06-08

**Authors:** Filipa Serrasqueiro, Ana Isabel Barbosa, Sofia A. Costa Lima, Salette Reis

**Affiliations:** 1 LAQV, REQUIMTE, Departamento de Ciências Químicas, Faculdade de Farmácia, Universidade do Porto, Rua de Jorge Viterbo Ferreira, 228, 4050-313 Porto, Portugal; 2 LAQV, REQUIMTE, Instituto de Ciências Biomédicas de Abel Salazar, Universidade do Porto, Rua de Jorge Viterbo Ferreira, 228, 4050-313 Porto, Portugal

**Keywords:** macrophage polarization, mannan, mannose, nano-immunotherapy, pro-inflammatory cytokines

## Abstract

Understanding how nanoparticles’ properties influence their cellular interactions is a bottleneck for improving the design of carriers. Macrophage polarization governs their active role in solving infections or tissue repair. To unravel the effect of carbohydrate-targeting mannose receptors on the macrophage surface, drug-free fucoidan/chitosan nanoparticles were functionalized using mannose (M) and mannan (Mn). Polyelectrolyte complex nanoparticles were obtained upon chitosan self-assembly using fucoidan. The functionalized nanoparticles were characterized in terms of their physicochemical characteristics, chemical profile, and carbohydrate orientation. The nanoparticles varied in size from 200 to 400 nm, were monodisperse, and had a stable negative zeta potential with a low aggregation tendency. The nonfunctionalized and functionalized nanoparticles retained their properties for up to 12 weeks. Cell viability and internalization studies were performed for all the designed nanoparticles in the THP-1 monocytes and THP-1-differentiated macrophages. The expression of the mannose receptor was verified in both immune cells. The carbohydrate-functionalized nanoparticles led to their activation and the production of pro-inflammatory cytokines interleukin (IL)-1β, IL-6, and tumour necrosis factor (TNF)-α. Both M- and Mn-coated nanoparticles modulate macrophages toward an M1-polarized state. These findings demonstrate the tailoring of these nanoplatforms to interact and alter the macrophage phenotype in vitro and represent their therapeutic potential either alone or in combination with a loaded drug for future studies.

## 1. Introduction

Macrophages are effector cells of the innate immune system, with crucial roles in homeostasis and host protection from pathogens [1]. Their ability to secrete a plethora of cytokines and growth factors mediate the resolution or production of inflammatory responses. In fact, the polarization state of macrophages is reversible and plastic, and is generally described as an M1/M2 dichotomy, which reflects their morphology and function/action [2]. M1 macrophages produce proinflammatory cytokines and reactive oxygen and nitrogen species and are thus actively involved in the phagocytosis of pathogens, while alternatively activated macrophages produce an M2 immune response and are involved in wound healing and tissue repair [3]. Macrophage activation toward the M1 or M2 polarization state occurs upon the activity of diverse factors, namely signaling molecules and their interaction with the cellular surface receptors. In particular, the mannose receptor (MR) or CD206 recognizes fucosylated and mannosylated glycoproteins, and is modulated by immunoglobulin receptors, cytokines, and pathogens [4]. This C-type lectin receptor is known to act in the clearance of endogenous glycoproteins and in pathogen recognition/antigen presentation [5]. Binding to MR triggers specific signaling pathways toward a tailored and robust immune response. The CD206 is a canonical marker that is expressed in the M2 macrophages responsible for M2 phenotypic functions of macrophages. Given the role of macrophages in diverse pathologies and the potential of their polarization state, developing delivery systems that are able to target and modulate macrophage function could represent a promising therapeutic tool.

Nanotechnology advances improve human health and wellbeing. The application of nanomedicine strategies allows for modulating the surface of a delivery system toward cellular targeting [6]. Surface coating the nanoparticles mediates this active targeting and may improve drug delivery and the therapeutic effect, as well as lowering side effects. Carbohydrate-functionalized delivery systems based on polymeric nanoparticles have been exploited as immunomodulatory agents [7,8,9,10]. The design of a polymeric nanoparticle coated with carbohydrates without the use of organic solvents or extensive synthetic chemistry would be an attractive tool for medical applications.

Natural polymers obtained from marine sources are attracting researchers’ attention given their abundance, low cost, low toxicity, and physicochemical resemblances to human tissue components [11]. Marine polysaccharides exhibit intrinsic properties of great applicability in drug delivery, namely, low immunogenicity, and the ability to be conjugated, complexed with proteins or other bioactive molecules, and even to produce stimuli-responsive systems [12]. Polyelectrolyte complexes of chitosan self-assembled with fucoidan form nanoparticles capable of delivering cargo through non-parenteral routes [13,14,15,16]. Oliveira and co-workers have demonstrated the effectiveness of targeting breast cancer cells upon fucoidan/chitosan nanoparticles functionalization with ErbB-2 antibodies [17]. Among several carbohydrate moieties that are already used in nanotechnology to tune the modulation of macrophages, mannose and mannan provide an excellent strategy toward enhancing the biocompatibility as well as favoring several therapeutics approaches that are already approved by the FDA [4].

In this context, we hypothesized that the interaction and uptake of mannose (M)- and mannan (Mn)-coated fucoidan/chitosan nanoparticles by macrophages could modulate their phenotype. Here, the functionalization of fucoidan/chitosan nanoparticles with carbohydrate moieties was obtained through a carbodiimide reaction. After physicochemical characterization, the cellular uptake of both types of carbohydrate-functionalized nanoparticles with macrophages was evaluated using a metabolic activity assay and flow cytometry. The ability of the nanoparticles to change macrophage polarization was demonstrated using a cytometric bead array. The gathered data elucidate on the modulation of macrophages through mannosylated-coated nanoparticles, which are a promising tool for nano-immunotherapy.

## 2. Results and Discussion

### 2.1. Characterization of the Fucoidan/Chitosan Nanoparticles

The polyelectrolyte complexed (PEC) nanoparticles, which were electrostatically self-assembled through Coulombic interactions between cationic chitosan and anionic fucoidan, were obtained at 5:1 (fucoidan/chitosan, F/C) ratio. The nanoparticles were characterized in terms of size, polydispersity index, and surface potential (Table 1).

The designed nanoparticles obtained through carbodiimide reaction exhibit a size range of 320–370 nm; thus, they are adequate for topical delivery [18]. The data collected with the carbohydrate-functionalized nanoparticles reveal a statistically significant increase in size in relation to the uncoated nanoparticles. The polydispersity and surface potential parameters were not affected by the functionalization process. The polydispersity index for all the nanoparticles is below 0.20, which suggests the presence of a homogenous nanoparticle population. The zeta potential values for the nanoparticles under study are around −30 mV, which indicates a high surface electric charge and, thus, a strong repulsion between the particles contributing to colloidal suspension stability. This negative value is related to the higher ratio of fucoidan in relation to the chitosan in the nanoparticle matrix, as previously described [14]. The F/C nanoparticles were loaded with FITC to perform cellular studies using fluorescent nanoparticles, and the similar properties of these nanoparticles were similar to the data collected for unloaded nanoparticles.

**Figure 1:**
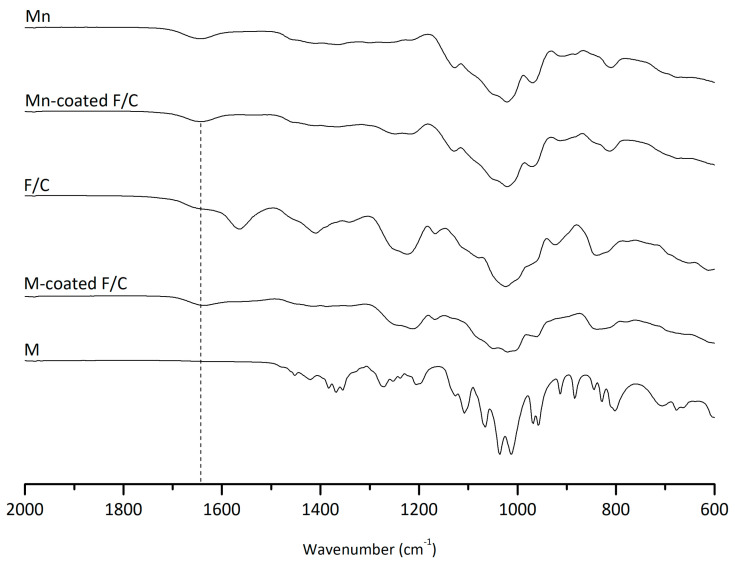
ATR-FTIR spectra to evaluate inter-molecular interactions between carbohydrates (Mn, M) and F/C nanoparticles.

The inter-molecular interactions of the carbohydrate-coated PEC nanoparticles were monitored via FTIR (Figure 1). The characteristic peaks of chitosan are evident at 1026 cm^−1^ of the C-O vibration; 1150 cm^−1^ of the asymmetric C-O-C vibration; the N-H bending between 1550 and 1640 cm^−1^; and a broad band between 1670 and 1820 cm^−1^, which may indicate C=O stretching from ester groups or overlapping carbonyl vibrations, though this does not confirm amide bond formation. Similarly, for fucoidan, the stretching of the sulfate groups (C-O-S) can be observed at 845 cm^−1^, and the S = O asymmetric stretching can be seen between 1160 and 1260 cm^−1^. As expected, the F/C PEC nanoparticles exhibit these characteristic peaks. The carbohydrate functionalization via the carbodiimide reaction consists of the activation of carboxylate functional groups, which can react with available hydroxyl moieties in mannose and mannan to form ester linkages, or alternatively promote non-covalent association to the nanoparticle surface. Figure 1 shows an increase in the C=O stretching region, which may be consistent with the formation of ester or amide-type bonds. Given the chemical functionalities present and data in the literature on fucoidan composition, the most probable mechanism involves ester bond formation, suggesting covalent or partially non-covalent attachment of carbohydrates.

### 2.2. Storage Stability of Fucoidan/Chitosan Nanoparticles

**Figure 2:**
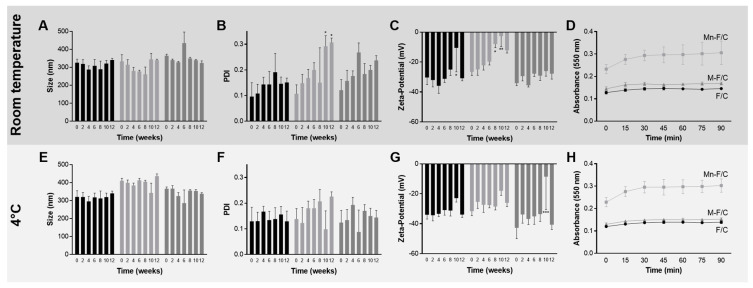
Storage stability of uncoated, Mn-coated, and M-coated F/C nanoparticles, black, light grey, and grey, respectively, at room temperature (top, dark grey) and 4 °C (bottom, grey). Parameter size (**A**,**E**), PDI (**B**,**F**), and zeta potential (**C**,**G**) and concanavalin A agglutination assay (**D**,**H**) were determined. Each result represents the mean ± standard deviation for n = 6, from two independent batches. * *p* < 0.05; ** *p* < 0.01; *** *p* < 0.001.

Figure 2 represents the physical stability of the F/C nanoparticles and carbohydrate-coated F/C nanoparticles during storage at room temperature and at 4 °C. The variation in the hydrodynamic diameter, polydispersity, and zeta potential were periodically evaluated. According to Figure 2B, at room temperature, no zeta potential variations were observed, reinforcing the long-term stability of the formulations conferred by the highly negatively charged F/C nanoparticle’s surface. The overall results show that uncoated, M-coated, and Mn-coated F/C nanoparticles are stable for at least 3 months since variations did not occur in the evaluated parameters, with the exception of Mn-coated F/C nanoparticles that, after 8-weeks stored at room temperature, became less negatively charged with evidence of aggregation tendency (PDI higher than 0.2, Figure 2B,C). When stored at 4 °C, no statistical differences were observed for the evaluated parameters over 12 weeks (Figure 2E–G). Previously, the uncoated-F/C nanoparticles also revealed storage stability after preparation when stored at room temperature [14]. Here, to assure the stability is maintained, the carbohydrate-coated PEC nanoparticles should be maintained at 4 °C.

During the storage period of the study, the presence/orientation of the carbohydrate-coated in the nanoparticles was evaluated via an agglutination assay. After exposure to concanavalin A, an increase in the absorbance at 550 nm was observed for the Mn-coated F/C nanoparticles in the same proportion for PEC nanoparticles stored at room temperature (Figure 2D) and at 4 °C (Figure 2H). The data are similar to those observed with the freshly prepared nanoparticles. On the contrary, the M-coated F/C nanoparticles do not exhibit an increase in the turbidity when incubated with concanavalin A. This behavior is similar to uncoated F/C nanoparticles, suggesting either the re-orientation or loss of mannose in the surface of the nanoparticles during storage at both temperature conditions under study. Thus, further studies were performed with freshly prepared M-coated F/C nanoparticles.

### 2.3. Cellular Evaluation

#### 2.3.1. Cell Viability Assessment

**Figure 3:**
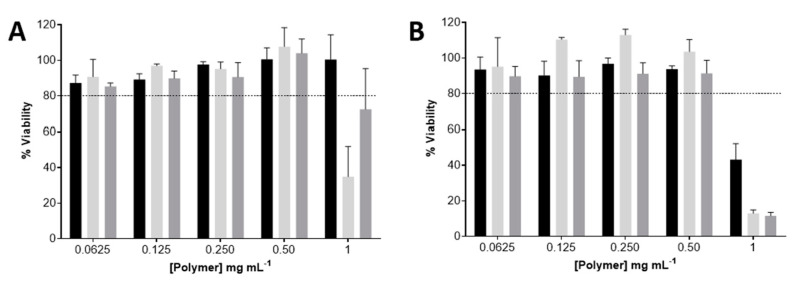
Viability of the uncoated, Mn-coated, and M-coated F/C nanoparticles, represented in black, light grey, and dark grey, respectively, evaluated via resazurin assay. (**A**) THP-1 monocytes; (**B**) THP-1-differentiated macrophages. Each result represents the mean ± standard deviation for n = 4 replicates of 3 assays. Horizontal dotted line indicates accepted limit of viability, 80%.

The THP-1 monocytes and THP-1-differentiated macrophages viability upon 24 h exposure to uncoated, M-coated, and Mn-coated PEC nanoparticles is presented in Figure 3. The uncoated-F/C nanoparticles resulted in a cell viability higher than 80% for the studied conditions, except for the highest concentration of M- and Mn-coated F/C nanoparticles. For the THP-1-differentiated macrophages, exposure to uncoated and carbohydrate-coated PEC nanoparticles lead to a loss in cell viability at 1 mg mL^−^^1^ in the polymer. To ensure the biocompatibility of the PEC nanoparticles in the THP-1 monocytes and THP-1-differentiated macrophages, cellular studies proceeded with the highest safe concentration (0.5 mg mL^−^^1^). Previous work reported the identical range of the biocompatibility of these F/C nanoparticles toward L929 fibroblasts [14].

#### 2.3.2. Cell–Nanoparticles Interaction

**Figure 4:**
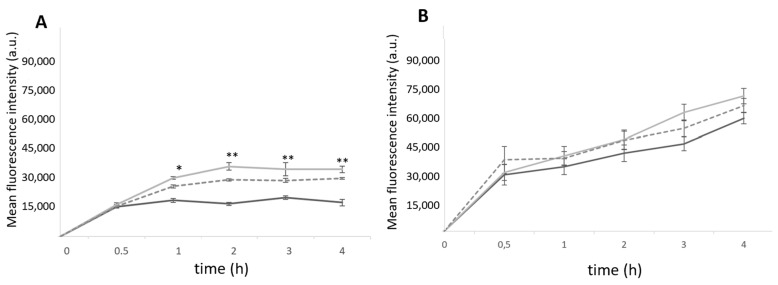
Uptake of FITC-loaded F/C nanoparticles at different incubation times, evaluated via flow cytometry. The uncoated, Mn-coated, and M-coated 5F1C nanoparticles, represented in black, light grey, and dark grey, respectively. (**A**) THP-1 monocytes; (**B**) THP-1-differentiated macrophages. Each result represents the mean ± standard deviation for n = 3 replicates of 3 assays. * *p* < 0.05; ** *p* < 0.01 compared uncoated F/C nanoparticles.

To understand the cell–nanoparticle interaction, the PEC nanoparticles were labelled with FITC and flow cytometric studies were conducted to assess the cellular uptake and its influence on the surface markers. The cellular uptake kinetics were evaluated up to 24 h for THP-1 monocytes and THP-1-differentiated macrophages (Figure 4).

The entry of nanoparticles into the THP-1 monocytes occurred within the first 30 min, for uncoated and carbohydrate-coated nanoparticles. After 2 h of incubation, the difference in uptake of the functionalized nanoparticles is noticed with a statistically significant higher fluorescence intensity compared to the nonfunctionalized nanoparticles. Among the functionalized ones, no statistically significant differences were observed. The Mn-coated F/C show higher internalization than the M-coated F/C. For the THP-1-differentiated macrophages, a time-dependent internalization was also observed without statistically significant differences between the uncoated and carbohydrate-coated nanoparticles within the first 4 h of incubation (Figure 4B). Thus, the results suggest that internalization occurs through a similar path for the uncoated and sugar-coated nanoparticles. To evaluate the possible molecular uptake mechanism of the F/C nanoparticles entry into the THP1-differentiated macrophages and monocytes, each cell type was incubated with uncoated and sugar-coated nanoparticles in the presence of inhibitors. After exposing the cells in the presence of nanoparticles at 4 °C, the endocytic-mediated internalization was confirmed as this energy-dependent process is blocked at this temperature. The clathrin-mediated endocytosis was confirmed for both uncoated and sugar-coated nanoparticles when the internalization decreased up to 40–50% in the presence of chlorpromazine and in a hypertonic condition (0.45 M sucrose). When the cells were preincubated with cytochalasin D, a macropinocytosis inhibitor, and then with each type of F/C nanoparticles under study, no inhibitory effect was observed in the internalization in relation to the nontreated cells (Figure S1). Taken together, this assessment indicates that the uptake of uncoated and sugar-coated F/C nanoparticles follows a clathrin-mediated endocytosis process as observed previously for carbohydrate-functionalized polymeric nanoparticles [8].

#### 2.3.3. Monocytes and Macrophage Polarization State

THP-1 monocytes and THP-1-differentiated macrophages have cell surface receptors/markers that may contribute to the internalization or to the modulation of the polarization state. The mannose receptor (MR, CD206) recognizes mannosylated glycoproteins and has the ability to engulf them [4,19,20]. On monocytes’ surfaces, CD11b plays a crucial role in the inflammatory responses, while the CD86 present on macrophages surface sets a marker of polarization toward a pro-inflammatory profile [8]. To evaluate the activation state of the THP-1 monocytes and THP-1-differentiated macrophages, the expression of these surface markers was analyzed, as well the secreted cytokines, upon exposure to the PEC nanoparticles under study.

**Figure 5:**
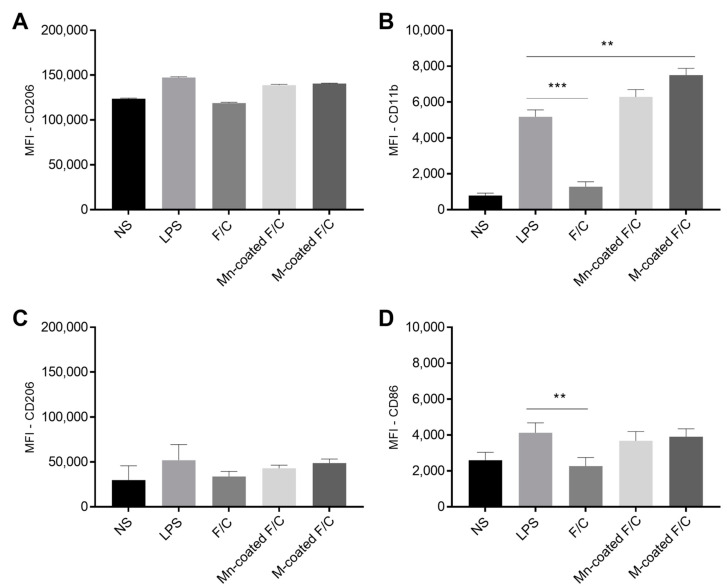
Analysis of the surface markers expression of THP-1 monocytes and differentiated macrophages. Non-stimulated (NS) and LPS-stimulated cells were used as negative and positive controls, respectively. Monocytes (**A**,**B**) and macrophages (**C**,**D**) were incubated with uncoated and sugar-coated F/C nanoparticles. Data represent the mean ± standard deviation (n = 3). MFI—mean fluorescence intensity. ** *p* < 0.01; *** *p* < 0.001 (in relation to LPS).

On the THP-1 monocytes and differentiated macrophages, the level of MR upon the incubation with the uncoated, Mn-coated, and M-coated F/C nanoparticles remain unchanged in relation to the LPS-stimulated cells and are similar to non-stimulated cells (Figure 5A, no significant statistical differences). However, CD11b surface marker expression is modulated in the THP-1 monocytes with the presence of carbohydrate-coated nanoparticles (Figure 5B). A significant increase in CD11b expression occurs in the presence of Mn- and M-coated F/C nanoparticles, similar to exposure to LPS, and has a statistically significant difference compared to the non-stimulated monocytes (*p* < 0.001), LPS (*p* < 0.05 and *p* < 0.01, respectively), and uncoated-F/C nanoparticles (*p* < 0.001). Thus, the carbohydrate-coated nanoparticles elicit the activation and maturation of monocytes as observed for the LPS stimulation. For the THP-1-differentiated macrophages, both functionalized F/C nanoparticles induced a higher expression of the co-stimulatory CD86 molecule as compared to the uncoated-F/C nanoparticles, which is similar to the LPS stimulation (Figure 5D). Others have reported an increase in surface markers expression for the carbohydrate-functionalized polymeric nanoparticles [8,21,22]. Macrophage surface modulation may also result in the secretion of cytokines that could trigger the T cells’ immune response. In fact, cytokine secretion is one of the most relevant indicators of macrophage polarization. The profile of cytokines produced by THP-1-differentiated macrophages with the uncoated, Mn-coated, and M-coated F/C nanoparticles was evaluated by determining the production of pro-inflammatory cytokines, including IL-1β, IL-6, and TNF-α (Table 2).

Both functionalized PEC nanoparticles led to the secretion of pro-inflammatory cytokines, IL-1β, IL-6, and TNF-α while the uncoated F/C nanoparticles did not. Of notice, the levels of IL-1β and TNF-α detected were higher than those secreted in the presence of LPS. The M-coated F/C nanoparticles resulted in the highest levels of the produced cytokines by the THP1-differentiated macrophages, but had no statistically significant difference from the Mn-coated F/C nanoparticles. Moreover, the untretead cells and macrophages incubated with each free sugar secrete negligible amounts of cytokines (Table S1). The levels of pro-inflammatory cytokines detected supports the immunomodulatory role of the nanoparticles toward a classical activation response, as previously described for other carbohydrate-functionalized nanoparticles’ action on macrophages [7,8,23] and dendritic cells [24,25].

Targeting macrophage receptors via the functionalization of drug-free polymeric nanoparticles represents a promising approach to modulate the immune system. Here, the sugar-coated F/C nanoparticles exhibit potential application in the case of intracellular pathogens, where a pro-inflammatory activity contributes to solving the infection. In fact, macrophages are crucial in the first line of defense against pathogens, acting as pattern recognition receptors for internalization and the degradation of several pathogens (e.g., Candida albicans, Leishmania donovani, and Mycobacterium tuberculosis) [4]. Future perspectives involve the application of these drug-free polymeric nanoparticles in an intracellular infection model per se or loaded with an anti-microbial agent to benefit from a possible synergistic effect.

## 3. Materials and Methods

### 3.1. Materials

The low-molecular-weight chitosan (molecular weight 50–190 KDa, degree of acetylation: 75–85%), fucoidan from Fucus vesiculosus ≥95% (molecular weight 50–190 kDa), D-(+)-mannose > 99% pure, mannan from Saccharomyces cerevisiae, phorbol 12-myristate 13- acetate (PMA) ≥ 99%, lipopolysaccharide (LPS) from Escherichia coli O111:B4, N-Hydroxysuccinimide (NHS), 1-ethyl-3-(-3-dimethylaminopropyl) carbodiimide hydrochloride (EDC), Concanavalin A from Canavalia ensiformis (Jack bean), magnesium chloride anhydrous ≥98%, and fluorescein isothiocyanate isomer I (FITC) were provided by Sigma-Aldrich (St. Louis, MO, USA). The acetic glacial acid was supplied by VWR International LLC (Radnor, PA, USA). The Dulbecco’s Phosphate Buffered Saline was acquired from Sigma Life Science (Poole, UK). For the cell culture, the Roswell Park Memorial Institute (RPMI) 1640 medium, Fetal Bovine Serum (FBS), penicillin-streptomycin (10,000 U/mL), and Hanks’ Balanced Salt Solution were acquired from Gibco^®^ (Invitrogen Corporation, Cheshire, UK). The resazurin sodium salt was purchased from Sigma-Aldrich (USA). The FITC anti-human CD206, APC anti-human CD11b, and PE anti-human CD86 were obtained from BioLegend^®^ (San Diego, CA, USA). The double-deionized water was provided by an ultra-pure water system (Easy 15 Water Purification System, Heal Force, Shanghai, China). The anti-human IL-6 Flex Set (Bead A7), anti-human IL-1β Flex Set (Bead D6), and TNF-α Flex Set (Bead C4) were used with the Human Soluble Protein Master Buffer Kit (Cytometric Brad Array (CBA), BD^TM^, Bioscience, Allschwil, Switzerland) to quantify the production of pro-inflammatory cytokines. The human leukemia monocyte THP1 cell line was obtained from the European Culture Collections (Salisbury, UK). All other chemicals and reagents used in the study were reagent grade or higher.

### 3.2. Methods

#### 3.2.1. Preparation of the Fucoidan/Chitosan Nanoparticles

The fucoidan/chitosan (F/C) nanoparticles were self-assembled through electrostatic interactions between the direct mixture of the polyanion and the polycation in aqueous solutions. Briefly, the fucoidan was dissolved in the double-deionized water to obtain a 5 mg mL^−^^1^ solution and the chitosan was dissolved in 1% (*v*/*v*) acid acetic solution at 1 mg mL^−^^1^. The F/C nanoparticles were prepared at a 5:1 ratio via electrostatic interactions, forming Polyelectrolyte complex nanoparticles under pulsed sonication (pulse-on 3 s and pulse-off 7 s, in a total of 30 s), at room temperature, using a probe sonicator (VCX130, Sonics and Material Vibra-CellTM with a CV-18 probe; 115 Newtown, CT, USA) to promote the nano-sized particles. After the self-assembly, the formulations were filtered using an 800 nm FilterBio^®^ Nylon Syringe Filter to remove any aggregations.

#### 3.2.2. Carbohydrate-Functionalization of the Fucoidan/Chitosan Nanoparticles

The functionalization with the mannan (Mn) and mannose (M) was achieved via a carbodiimide chemical reaction. The EDC (30 µg mL^−^^1^ in PBS pH 5) and NHS (100 µL mL^−^^1^) were added to 9 mg of the F/C nanoparticles in 4.5 mL and the mixture was then stirred at room temperature overnight. After this step, 18 mg of Mn and M were added to the nanoparticles, and the mixture were left under stirring at room temperature overnight.

The Mn- and M-coated F/C nanoparticles were dialyzed (dialysis bag; MWCO 12–14 kD, Dialysis Membrane Spectrum Laboratories) against 250 mL of double-deionized water, at room temperature, for 5 h to remove any unreacted mannan or mannose or other impurities.

The FITC-loaded F/C nanoparticles were prepared as previously described, by the addition of 45 µL of FITC (2 mg mL^−^^1^) to the mixture of fucoidan and chitosan, prior to the sonication step. Further coating with carbohydrate moieties was also employed to the FITC-loaded F/C nanoparticles when needed.

#### 3.2.3. Characterization of Fucoidan/Chitosan Nanoparticles

##### Determination of Particle Size, Polydispersity Index, and Surface Potential

The functionalized and non-functionalized nanoparticles were characterized in terms of mean size and size distribution (polydispersity index, PDI) using dynamic light scattering (DLS) in a Particle Size Analyzer (Brookhaven Instruments Corporation; Software: Particle Sizing v.5 Brookhaven Instruments; Holtsville, NY, USA). For each condition, 6 runs of 2 min were performed, operating at a scattering angle of 90° at 25 °C. All the samples had a suitable concentration for measurement (300–500 kcps). The zeta potential of the developed nanoparticles was measured using an electrode and a Zeta Potential Analyzer (ZetaPALS, Brookhaven Instruments Corporation, Software: PALS Zeta Potential Analyzer v.5, Brookhaven Instruments; Holtsville, NY, USA) operating at a scattering angle of 90°. For each assay, 6 runs of 10 cycles were performed at 25 °C.

##### Analysis of Chemical Interactions via Fourier Transform Infrared Spectroscopy Analysis

Prior to the Fourier Transform Infrared (FTIR) spectroscopy analysis, the nanoparticle solutions were lyophilized, upon freezing the sample. Briefly, all the samples were kept overnight in a −80 °C freezer (Deep Freezer, GFL^®^, Germany), and then lyophilized in a freeze drier (LyoQuest −85 plus v.407, Telstar) for 72 h while continuously kept at −75 °C and 0.40 mbar of pressure.

After the lyophilization process, the functionalized and non-functionalized nanoparticles were analyzed using a FTIR Spectrophotometer (Perkin Elmer, FT-IR Spectrometer Frontier) equipped with a diamond crystal. The reference compounds and lyophilized nanoparticles were placed directly into the attenuated total reflectance (ATR) compartment for analysis at room temperature, after a background run with an empty ATR accessory. The obtained spectra were a result of the 32 combined scans recorded between 4000 and 600 cm^−^^1^, with a spectra resolution of 4 cm^−^^1^.

##### Agglutination Assay

To verify the orientation of the carbohydrate moieties associated with the surface of the F/C nanoparticles, an agglutination assay was performed. For this effect, 500 µL of nanoparticles were diluted in 500 µL of double-deionized water and centrifuged at 10,000× *g* for about 20 min. The supernatant was removed, and the pellet was re-suspended in 1 mL of PBS, 10 mM at pH 7. A volume of 200 µL of each type of nanoparticles under study were incubated with 50 µL of concanavalin A solution (1 mg mL^−^^1^). The time-dependent increase in the turbidity at 405 and 550 nm was monitored spectrophotometrically (Sinergy HT, BioTek^®^; Bedfordshire, UK) for 90 min at 25 °C.

#### 3.2.4. Storage Stability Studies

In order to evaluate the storage stability of the functionalized and non-functionalized nanoparticles through time, a study was conducted over 12 weeks by evaluating the particle size, PDI, and zeta potential using the characterization techniques previously mentioned. The nanoparticles were stored in glass vials, at room temperature and at 4 °C.

#### 3.2.5. Cellular Assays

##### THP-1 Monocytes and Differentiated Macrophages Cultures

The human THP-1 monocytes cells were cultured at 37 °C in a 5% CO_2_ atmosphere (Unitherm CO_2_ Incubator 3503 Uniequip; Planegg, Germany) in the Gibco Roswell Park Memorial Institute (RPMI) 1640 medium supplemented with 10% (*v*/*v*) of Fetal Bovine Serum (FBS) and 1% (*v*/*v*) Penicillin-Streptomycin. The cells were routinely cultured in 25 cm^2^ culture flasks and passed every 2–3 days. 

The THP-1 monocytes were differentiated into macrophages in 24- or 96-well plates, after 18 h in the presence of 20 ng mL^−^^1^ PMA, followed by 24 h with fresh supplemented culture medium. 

##### Cell Viability Assay

The effect of the developed F/C nanoparticles on the monocytes and differentiated macrophages was evaluated using the resazurin assay. For the THP-1 monocytes, the cells were seeded in a 96-well plate at a density of 10^5^ cells/well. The initial concentration (2 mg mL^−^^1^) of each nanoparticle type was diluted in supplemented culture media to the concentrations range of 0.06–1.00 mg mL^−^^1^ and incubated with cells in a final volume of 90 µL. The positive control untreated cells were used, while the negative control cells were exposed to 1% (*v*/*v*) Triton X-100TM. The THP-1 monocytes were incubated with all produced nanoparticle types for 24 h at 37 °C in a 5% CO_2_ atmosphere. Then, 10 µL of resazurin solution at 440 µM were added, followed by incubation for ca. 3 h at 37 °C in 5% CO_2_ atmosphere. An identical procedure was performed for the THP1-differentiated macrophages.

The metabolic activity of the cells was quantified via fluorimetry using a microplate reader (Cytation 3, BioTek^®^; Bedfordshire, UK); after excitation at wavelength 528 nm, the fluorescence emission was obtained at 590 nm. The results were expressed as the percentage of the metabolic activity of the treated cells with different concentrations of nanoparticles relative to the untreated cells.

##### Cellular Uptake Study

For the cellular uptake assay, all the nanoparticles under study were prepared to contain FITC. A non-toxic concentration of nanoparticles (0.5 mg mL^−^^1^) was selected to perform the study in the THP-1 monocytes and THP-1 differentiated macrophages. Briefly, the monocytes seeded in 24-well plates at a density of 2 *×* 10^5^ cells/well in the supplemented RPMI and incubated with FITC-loaded F/C nanoparticles at 37 °C and a 5% CO_2_ atmosphere. After specific times, the solution was centrifuged at 300× *g* for 5 min, using an Allegra^®^X-15R centrifuge (Beckman Coulter, Pasadena, CA, USA), and then the cells were recovered in 100 µL of PBS. 

The THP-1-differentiated macrophages were seeded in 24-well plates at a density of 6 × 10^5^ cells/well in supplemented RPMI and were incubated with FITC-loaded F/C nanoparticles at 37 °C and a 5% CO_2_ atmosphere. After specific times, the cells were washed with PBS pH 7.4 to remove the noninternalized nanoparticles and detached with 0.25% trypsin-EDTA. The solution was centrifuged at 300× *g* for 5 min, using an Allegra^®^X-15R centrifuge (Beckman Coulter, Pasadena, CA, USA), and then the cells were recovered in 100 µL of PBS. Prior to the flow cytometry analysis, 1 µL of trypan blue was added to eliminate the fluorescence of the nanoparticles that did not internalize the cells, and 1 µL propidium iodide, to exclude the dead cells. For each sample, a minimum of 10,000 events were recorded, under FL1 in a flow cytometer (Accuri^TM^, BD Biosciences, Erembodegem, Belgium).

##### Evaluation of Secreted Cytokines and Cell Surface Markers

In a 24-well plate, the THP-1 monocytes (2 *×* 10^5^ cells/well) or THP-1-differentiated macrophages (6 *×* 10^5^ cells/well) were exposed for 20 h to a non-toxic dosage of all produced nanoparticles (viability > 80%), and the wells for positive controls were stimulated with LPS (1.0 µg mL^−^^1^) 6 h before the analysis. Then, the suspension was centrifuged at 300× *g* for 5 min, using an Allegra^®^X-15R centrifuge (Beckman Coulter, Pasadena, CA, USA), and the supernatants were harvested and stored at −20 °C until analysis. For the cytokine analysis, the Cytometric Bead Array Flex Set (BD, San Jose, CA, USA) was used according to the manufacturer’s instructions.

For the cellular surface markers evaluation, upon incubation with each developed nanoparticle, the cells were recovered via centrifugation and labelled with CD206-FITC, CD11b-APC (monocytes) and CD86-PE (macrophages), following manufacturer’s instructions. For each sample, a minimum of 10,000 events were recorded in the flow cytometer (AccuriTM, BD Biosciences, Erembodegem, Belgium).

#### 3.2.6. Statistical Analysis

The statistical analysis was performed using the GraphPad Prism Software (Version 8.4.3 for Windows; GraphPad Software Inc., San Diego, CA, USA). The one-way and two-way ANOVA were used to assess the differences between the formulations.

## 4. Conclusions

Mannosylated drug delivery systems have been reported to interact with macrophages using transmembrane receptors. The exploitation of drug-free delivery systems and their cellular interplay, namely, macrophage activation and immunomodulatory potential, is still limited. Here, the polarization of human THP-1-differentiated macrophages via drug-free fucoidan/chitosan nanoparticles functionalized with mannose or mannan was achieved. Cellular uptake occurred within 1 h, via clathrin-mediated endocytosis and triggered M1 polarization, according to the expressed surface markers and secreted pro-inflammatory cytokines. Both mannose- and mannan-coating carbohydrates led to classically activated macrophages, but not with non-functionalized nanoparticles. Regarded together, the data show the ability of these drug-free nanoparticles to change the human macrophages’ phenotype in vitro, thus indicating their potential as therapeutic tools for treating intracellular infections or even cancer.

## Figures and Tables

**Table 1:** Physicochemical characterization of uncoated, Mn- and M-coated F/C nanoparticles using DLS measurements.

Nanoformulation	Size (nm)	PDI	ζ-Potential (mV)
F/C	323 ± 28	0.112 ± 0.057	−32 ± 4
Mn-coated F/C	372 ± 8 *	0.123 ± 0.041	−29 ± 4
M-coated F/C	365 ± 9 *	0.122 ± 0.044	−38 ± 7

Mn—manan, M—mannose, F/C—fucoidan/chitosan, PDI—polydispersity index. Data expressed as mean ± standard deviation, from four independent batches. * *p* < 0.05.

**Table 2:** THP-1-differentiated macrophages secreted cytokines upon exposure to uncoated and carbohydrate-coated F/C nanoparticles.

	IL1β (pg/mL)	IL-6 (pg/mL)	TNF-α (pg/mL)
LPS	575 ± 21	2105 ± 81	1545 ± 94
F/C	250 ± 16 ***	1340 ± 94 ***	1145 ± 77 *
Mn-coated F/C	658 ± 37 *	2157 ± 78	1845 ± 102 *
M-coated F/C	705 ± 43 *	2208 ± 56	1922 ± 96 *

Data represent mean ± standard deviation (n = 3) * *p* < 0.01; *** *p* < 0.001 in relation to LPS treatment.

## Data Availability

Data are contained within the article.

## Supplementary Materials

The supporting information can be downloaded at: https://www.mdpi.com/article/10.3390/ijms24129908/s1.
