# Correction: Serrasqueiro et al. Targeting the Mannose Receptor with Functionalized Fucoidan/Chitosan Nanoparticles Triggers the Classical Activation of Macrophages. *Int. J. Mol. Sci.* 2023, *24*, 9908

**DOI:** 10.3390/ijms262311634

**Published:** 2025-12-01

**Authors:** Filipa Serrasqueiro, Ana Isabel Barbosa, Sofia A. Costa Lima, Salette Reis

**Affiliations:** 1 LAQV, REQUIMTE, Departamento de Ciências Químicas, Faculdade de Farmácia, Universidade do Porto, Rua de Jorge Viterbo Ferreira, 228, 4050-313 Porto, Portugal; up201508747@edu.ff.up.pt (F.S.); up200800307@edu.ff.up.pt (A.I.B.); 2 LAQV, REQUIMTE, Instituto de Ciências Biomédicas de Abel Salazar, Universidade do Porto, Rua de Jorge Viterbo Ferreira, 228, 4050-313 Porto, Portugal

There was an error in the original publication [1]. While EDC/NHS coupling was employed, the confirmation of amide bond formation with mannose/mannan cannot be definitively concluded from FTIR analysis alone. Given the chemical structure of mannose/mannan (which lacks primary amine groups), ester bond formation or non-covalent interactions are more plausible. Therefore, the term ‘amide bond’ has been corrected to ‘ester bond or non-covalent interaction’ throughout the manuscript where applicable.

A correction has been made to Section 2.1 Characterization of the Fucoidan/Chitosan Nanoparticles, in the third paragraph:

The inter-molecular interactions of the carbohydrate-coated PEC nanoparticles were monitored via FTIR (Figure 1). The characteristic peaks of chitosan are evident at 1026 cm^−1^ of the C-O vibration; 1150 cm^−1^ of the asymmetric C-O-C vibration; the N-H bending between 1550 and 1640 cm^−1^; and a broad band between 1670 and 1820 cm^−1^, which may indicate C=O stretching from ester groups or overlapping carbonyl vibrations, though this does not confirm amide bond formation. Similarly, for fucoidan, the stretching of the sulfate groups (C-O-S) can be observed at 845 cm^−1^, and the S = O asymmetric stretching can be seen between 1160 and 1260 cm^−1^. As expected, the F/C PEC nanoparticles exhibit these characteristic peaks. The carbohydrate functionalization via the carbodiimide reaction consists of the activation of carboxylate functional groups, which can react with available hydroxyl moieties in mannose and mannan to form ester linkages, or alternatively promote non-covalent association to the nanoparticle surface. Figure 1 shows an increase in the C=O stretching region, which may be consistent with the formation of ester or amide-type bonds. Given the chemical functionalities present and data in the literature on fucoidan composition, the most probable mechanism involves ester bond formation, suggesting covalent or partially non-covalent attachment of carbohydrates.

The authors state that the scientific conclusions are unaffected. This correction was approved by the Academic Editor. The original publication has also been updated.
