# Supplementary material for: Targeting the Mannose Receptor with Functionalized Fucoidan/Chitosan Nanoparticles Triggers the Classical Activation of Macrophages
Source: Int J Mol Sci. 2023 Jun 8;24(12):9908. doi: 10.3390/ijms24129908 (PMC10298651; doi:10.3390/ijms24129908)
Supplement: Supplementary file 1 [file ijms-24-09908-s001.zip › ijms-2428518-supplementary.pdf]

Targeting mannose receptor with functionalized fucoidan/chitosan nanoparticles triggers the classical activation of macrophages

Filipa Serrasqueiro[1], Ana Isabel Barbosa[1], Sofia A. Costa Lima[2,*], Salette Reis[1,*]

Supplementary data

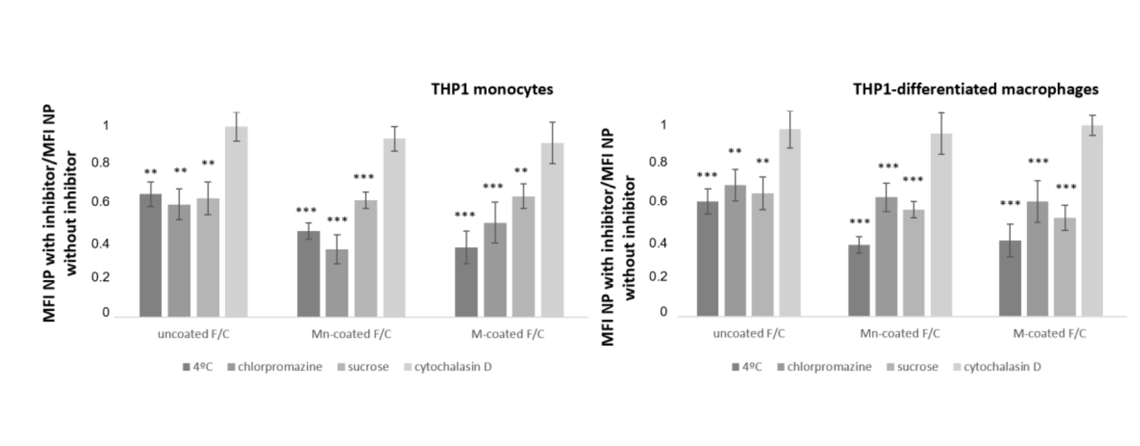

Figure S1. Mechanism of internalization of uncoated and sugar-coated F/C nanoparticles. Effect of inhibitors of clathrin-mediated endocytosis (4°C; chlorpromazine and sucrose) and of macropinocytosis (cytochalasin D) upon 1 hour of incubation with THP1 monocytes and differentiated macrophages. MFI – mean fluorescence intensity; NP – nanoparticle; F/C – fucoidan/chitosan; Mn – mannan; M – mannose. ** $p < 0.01$; *** $p < 0.001$.

Table S1. THP-1-differentiated macrophages secreted cytokines

	**IL1β** (pg/mL)	**IL-6** (pg/mL)	**TNF-α (**pg/mL)
Untreated cells	55 ± 9	214 ± 11	235 ± 9
Mn	63 ± 6	240 ± 19	215 ± 7
M	65 ± 3	228 ± 6	198 ± 9

Data represents mean ± standard deviation (n=3)
